# Genomic Investigation of *Salmonella* Isolates Recovered From a Pig Slaughtering Process in Hangzhou, China

**DOI:** 10.3389/fmicb.2021.704636

**Published:** 2021-07-08

**Authors:** Beibei Wu, Abdelaziz Ed-Dra, Hang Pan, Chenghang Dong, Chenghao Jia, Min Yue

**Affiliations:** ^1^Zhejiang Provincial Center for Disease Control and Prevention, Hangzhou, China; ^2^Hainan Institute of Zhejiang University, Sanya, China; ^3^Department of Veterinary Medicine, Institute of Preventive Veterinary Sciences, Zhejiang University College of Animal Sciences, Hangzhou, China; ^4^State Key Laboratory for Diagnosis and Treatment of Infectious Diseases, National Clinical Research Center for Infectious Diseases, National Medical Center for Infectious Diseases, The First Affiliated Hospital, College of Medicine, Zhejiang University, Hangzhou, China; ^5^Zhejiang Provincial Key Laboratory of Preventive Veterinary Medicine, Hangzhou, China

**Keywords:** *Salmonella*, antimicrobial resistance, plasmid replicons, virulence factors, pig slaughterhouse, whole genome sequencing

## Abstract

The pig industry is the principal source of meat products in China, and the presence of pathogens in pig-borne meat is a crucial threat to public health. *Salmonella* is the major pathogen associated with pig-borne diseases. However, route surveillance by genomic platforms along the food chain is still limited in China. Here, we conducted a study to evaluate the dynamic prevalence of *Salmonella* in a pig slaughtering process in Hangzhou, Zhejiang Province, China. Fifty-five of 226 (24.37%) samples were positive for *Salmonella*; from them, 78 different isolates were selected and subjected to whole genome sequencing followed by bioinformatics analyses to determine serovar distribution, MLST patterns, antimicrobial resistance genes, plasmid replicons, and virulence factors. Moreover, phenotypic antimicrobial resistance was performed using the broth dilution method against 14 antimicrobial agents belonging to 10 antimicrobial classes. Our results showed that samples collected from the dehairing area (66.66%) and the splitting area (57.14%) were the most contaminated. Phenotypic antimicrobial resistance classified 67 of 78 isolates (85.90%) as having multidrug resistance (MDR), while the highest resistance was observed in tetracycline (85.90%; 67/78) followed by ampicillin (84.62%; 66/78), chloramphenicol (71.80%; 56/78), and nalidixic acid (61.54%; 48/78). Additionally, serovar prediction showed the dominance of *Salmonella* Typhimurium ST19 (51.28%; 40/78) among the 78 studied isolates, while plasmid prediction reported the dominance of IncHI2A_1 (20.51%; 16/78), followed by IncX1_1 (17.95%; 14/78) and IncHI2_1 (11.54%; 9/78). Virulence factor prediction showed the detection of *cdtB* gene encoding typhoid toxins in two *Salmonella* Goldcoast ST358 and one *Salmonella* Typhimurium ST19, while one isolate of *Salmonella* London ST155 was positive for genes encoding for the siderophore “yersiniabactin” and the gene *senB* encoding for enterotoxin production. From this study, we conclude that pig slaughterhouses are critical points for the dissemination of virulent and multidrug-resistant *Salmonella* isolates along the food chain which require the implementation of management systems to control the critical points. Moreover, there is an urgent need for the implementation of the whole genome sequencing platform to monitor the emergence of virulent and multidrug-resistant clones along the food chain.

## Introduction

Salmonellosis is a global zoonotic disease, caused by *Salmonella* and characterized by self-limited gastroenteritis in immunocompetent adults, in which typical symptoms like diarrhea, fever, abdominal cramps, and vomiting occur between 6 and 72 h (usually 12–36 h) after ingestion of bacteria and the illness lasts from 2 to 7 days [World Health Organisation (WHO), 2018]. It might also cause severe invasive infection, particularly in immunocompromised patients (Deen et al., 2012; Xu X. et al., 2020). Recently, it was estimated that *Salmonella* was responsible for about 180 million (9%) of the diarrheal illnesses that occur globally each year, causing about 298,000 deaths (41%) of all diarrheal disease-associated deaths (Besser, 2018). In China, a study based on the literature review estimated that the incidence of nontyphoidal salmonellosis was 626.5 cases per 100,000 persons (Mao et al., 2011; Xu Y. et al., 2020). Moreover, it has been reported that *Salmonella* was responsible for approximately 70∼80% of foodborne pathogenic outbreaks in China (Jun et al., 2007).

*Salmonella* spp. are Gram-negative rod-shaped bacteria, facultatively anaerobic, and belong to the family Enterobacteriaceae. To date, more than 2,600 serovars have been described among *Salmonella* species; among them, only a few serovars were mostly linked to human and/or animal infections, including Typhimurium and Enteritidis (so-called majority serovars) for human infections (Xu X. et al., 2020), Gallinarum and Pullorum for poultry infections (Xu Y. et al., 2020), Dublin for cattle infections (Paudyal et al., 2019), and Choleraesuis and Typhisuis for pig infections (Boyen et al., 2008; Asai et al., 2010). Generally, animal farms are considered natural reservoirs of *Salmonella*, especially poultry and pigs (Li et al., 2013; Zhou et al., 2017; Xu Y. et al., 2020). *Salmonella* could colonize the digestive tract of animals and are excreted in feces and spread into the environment (Kagambèga et al., 2013; Bonardi, 2017; Jiang et al., 2019), then transmitted to humans *via* the food chain (Ed-Dra et al., 2018; Wang et al., 2019; Wilson et al., 2020; Liu et al., 2021). Therefore, several studies have reported the presence of *Salmonella* in foods of animal origin, especially meat products (Ed-Dra et al., 2017; Jiang et al., 2021; Liu et al., 2021).

Pork meat is considered the most frequently contaminated food and the major source of *Salmonella* infections in humans (Bonardi, 2017; Wilson et al., 2020). In fact, pig farms seem to be a suitable environment for the replication and the persistence of *Salmonella* (Lettini et al., 2016; Bonardi, 2017; Vico et al., 2020). However, the slaughtering process which is located downstream of the pig-breeding process and upstream of pork sales is a critical step in determining the contamination/decontamination of animal carcasses and thus the meat products (Zhou et al., 2018). Moreover, the application of good hygienic practices in slaughterhouses has great importance and could participate in reducing the prevalence of *Salmonella* in the final meat products (Rahkio and Korkeala, 1996; Biasino et al., 2018). During the slaughtering process, animals pass through different processing stages with complicated manipulations (Zhou et al., 2018). However, since pigs are considered reservoirs of pathogens, they could contaminate/cross-contaminate the carcasses or muscle tissues during the slaughtering process. In fact, it has been demonstrated that slaughter practices, such as splitting the head and incising tonsils, were associated with higher levels of hygiene indicator bacteria and *Salmonella* in pig carcasses (Biasino et al., 2018). Therefore, the surveillance of *Salmonella* along the slaughtering process and its environment is with a high priority to determine the key points that are responsible for the contamination of carcasses and the final meats.

Recently, whole genome sequencing followed by bioinformatics analysis was considered as a cost-effective method for the diagnosis and characterization of foodborne pathogens (Biswas et al., 2020; Liu et al., 2020, 2021; Yu et al., 2020). As proof-of-concept, we conduct a study in a pig slaughterhouse in Hangzhou (Zhejiang Province, China), to obtain *Salmonella* isolates from different sources. The recovered strains were subjected to whole genome sequencing followed by *in silico* analysis to determine serovar distribution, multilocus sequence types, plasmid replicons, antimicrobial resistance, and virulence genes. Moreover, phenotypical antimicrobial resistance was investigated by the broth dilution method and compared with genotypical resistance.

## Materials and Methods

### Sample Collection and Characterization of *Salmonella*

The present study was conducted in Linpu Pig Slaughterhouse in Xiaoshan, Hangzhou (China). The capacity of the studied slaughterhouse was approximately 1,000 pigs per day. A sampling visit was organized during December 2018 allowing the collection of 226 samples from different origins (pig carcasses, swab samples, environmental samples, equipment samples, operator samples, intestinal content samples, hepatobiliary samples, and sewer samples) along the slaughtering process of pigs (Table 1). The sampling method was in accordance with those described in previous studies (Cai et al., 2016; Zhou et al., 2017). The isolation of *Salmonella* was performed from different samples according to the protocols described previously (Jiang et al., 2019; Liu et al., 2021). Then, molecular confirmation of presumptive isolates was carried out by the amplification of *invA* gene according to the protocol previously described (Zhu et al., 2015; Liu et al., 2021).

**TABLE 1:** Sampling design and prevalence of *Salmonella* from different sources.

Sources	No. of samples	No. of positive samples	Percentage of contamination
**Slaughtering process**			
Live animal area	15	2	13.33%
Bleeding area	6	1	16.66%
Washing area	4	0	0%
Scalding area	10	0	0%
Dehairing area	6	4	66.66%
Cleaning the beating area	4	2	50%
Splitting area	14	8	57.14%
Clean area after splitting	4	1	25%
Carcass trimming area	7	1	14.58%
Meat inspection area	7	1	14.58%
Disinfection and precooling area	6	2	33.33%
**Others**			
Sewer outlet	6	2	33.33%
Bile samples	30	7	23.33%
Intestinal samples	47	19	40.42%
Liver samples	30	4	13.33%
Mesenteric lymph node samples	30	1	3.33%
Total	226	55	24.37%

Among the 226 collected samples, 55 were positive for the presence of *Salmonella*. However, since some samples present more than one presumptive isolate, we decided to select 78 different *Salmonella* isolates that show differences in morphological and biochemical criteria for genome sequencing and analysis (Table 2). Serotyping of the PCR confirmed *Salmonella* isolates were performed according to White–Kauffmann–Le Minor scheme by slide agglutination method to define O and H antigens using commercial antisera (SSI Diagnostica, Hillerød, Denmark).

**TABLE 2:** Distribution of the studied *Salmonella* isolates according to sampling sources.

Sampling sources	No. of isolates	Grouping samples	No. of isolates
Inventory area	4 (5.13%)	Carcass swabs before splitting (CSBS)	19 (24.36%)
Bloodletting area	3 (3.85%)		
Dehairing area	11 (14.10%)		
Cleaning the beating area	1 (1.28%)		
Splitting area	12 (15.38%)	Carcass swabs after splitting (CSAS)	15 (19.23%)
Carcass trimming area	1 (1.28%)		
Meat inspection area	2 (2.56%)		
Bile samples	11 (14.10%)	Hepatobiliary samples (HS)	16 (20.51%)
Liver samples	5 (6.41%)		
Stool sample	22 (28.20%)	Fecal samples (FS)	22 (28.20%)
Sewer mouth sample	6 (7.69%)	Sewer samples (SS)	6 (7.69%)
Total	78 (100%)	Total	78 (100%)

### Genomic DNA Extraction

All the obtained *Salmonella* isolates (*n* = 78) were selected for genomic DNA extraction according to the protocol described previously (Liu et al., 2021). Briefly, a broth culture of each *Salmonella* isolate was prepared by inoculation of a pure colony in Luria–Bertani broth followed by incubation at 37°C under 180 rpm shaking conditions. Then, DNA extraction was conducted by using TIANamp bacteria DNA kit (Tiangen Biotech, Beijing, China) according to the instructions of the manufacturer. The quantification of the extracted DNA was performed by the Qubit 2.0 Fluorometer (Invitrogen, Carlsbad, CA, United States), as per the instructions of the manufacturer.

### Genomic Sequencing and Bioinformatic Analysis

The genomic DNA library was constructed using NovaSeq XT DNA library construction kit (Illumina, San Diego, CA, United States, No: FC-131-1024), followed by genomic sequencing using Illumina NovaSeq Platform with NovaSeq 6000 SP Reagent Kit (300 cycles). The raw sequence reads were checked for quality and assembled using SPAdes v3.12.0 (Bankevich et al., 2012). Virulence gene prediction was conducted based on the virulence factors database (VFDB) (Chen et al., 2005). Moreover, *in silico* serotyping of *Salmonella* strains was performed by the SISTR web tool, whereas sequence types, antimicrobial resistance genes (ARG), and plasmid replicons were detected using the assemblies of the samples on the in-house Galaxy platform (Afgan et al., 2016), in combination with mlst v2.16.1^1^ and abricate v0.8 (Zankari et al., 2012), including the CGE ResFinder database (updated on February 19, 2021) with a similarity cutoff of 90% for ARG and PlasmidFinder database (updated on February 19, 2021) with a similarity cutoff of 95% (Carattoli et al., 2014).

### Phenotypic Antimicrobial Resistance Testing

The antimicrobial resistance of the isolated *Salmonella* strains was evaluated phenotypically by the broth dilution method to determine the minimum inhibitory concentration (MIC) of a panel of 14 antimicrobial agents belonging to 10 antimicrobial classes according to the protocol described previously (Jiang et al., 2021; Liu et al., 2021). The obtained results were interpreted according to the recommendation of the Clinical Laboratory Standard Institute guidelines (CLSI, 2017). The tested antimicrobial agents were as follows: penicillins (ampicillin: AMP, 0.25–128 μg/ml), β-lactamase inhibitors (amoxicillin/clavulanic acid: AMC, 0.125/0.062–128/64 μg/ml), cephems (ceftiofur: CF, 0.125–128 μg/ml; cefoxitin: CX, 0.125–128 μg/ml), aminoglycosides (gentamicin: GEN, 0.031–64 μg/ml; kanamycin: KAN, 0.25–128 μg/ml; streptomycin: STR, 1–128 μg/ml), tetracyclines (tetracycline: TET, 0.062–128 μg/ml), fluoroquinolones (ciprofloxacin: CIP, 0.015–16 μg/ml; nalidixic acid: NAL, 0.5–128 μg/ml), folate pathway inhibitors (trimethoprim/sulfamethoxazole: TST, 0.25/4.75–32/608 μg/ml), polypeptides (colistin: COL, 0.031–64 μg/ml), macrolides (azithromycin: AZI, 0.25–128 μg/ml), and phenicols (chloramphenicol: CHL, 0.5–128 μg/ml). *Escherichia coli* ATCC 25922 and *Pseudomonas aeruginosa* ATCC 27853 were used as the quality control strains to validate the antimicrobial susceptibility testing. However, strains showing a decrease in susceptibility (intermediate) were merged with resistant strains for ease of analysis, and the multidrug-resistant (MDR) strains were defined by resistance to at least three antimicrobial classes.

### Data Analysis

GraphPad Prism 8 software (San Diego, CA, United States) was used for data analysis and generation of the figures. For antimicrobial susceptibility testing, the results of intermediate susceptibility were merged with resistance. Then, each phenotypically antimicrobial susceptibility test result (resistant or susceptible) was compared with the detection (presence or absence) of the corresponding resistance gene by *in silico* analysis. The isolates that are positive for at least one antimicrobial resistance gene among an antimicrobial class were considered as resistant to the corresponding antimicrobial class. The coherent results group together the isolates that are resistant or susceptible for both phenotypical and genotypical results. However, the incoherent results correspond to the isolates that are phenotypically resistant and genotypically susceptible or phenotypically susceptible and genotypically resistant for an antimicrobial agent. The percentage of incoherence corresponds to the difference between the results obtained by phenotypical and genotypical tests for each antimicrobial agent.

## Results

### *Salmonella* Prevalence, MLST Pattern, and Serovar Distribution

The results obtained in this study showed that 55 of 226 (24.37%) samples were contaminated by *Salmonella* (Table 1). According to the sampling points along the pig slaughtering process, our results showed that the samples collected from the dehairing area were the most contaminated (66.66%), followed by those collected from the splitting area (57.14%). However, samples collected from the washing area and scalding area were not contaminated (Table 1). Additionally, from the 55 samples, 78 different *Salmonella* isolates were obtained, purified, and subjected to whole genome sequencing. The genomic prediction of serovars and MLST patterns showed the distribution of five different serovars and six MLST patterns, namely, Typhimurium ST19 (*n* = 40), Typhimurium ST34 (*n* = 14), London ST155 (*n* = 14), Rissen ST469 (*n* = 7), Goldcoast ST358 (*n* = 2), and Derby ST40 (*n* = 1) (Table 3). Additionally, serotyping performed by *in silico* analysis and slide agglutination methods provided the same results.

**TABLE 3:** Allelic profiles, serogroups, serovars, and MLST patterns of *Salmonella* isolated from different sources.

Serogroup	Serovar	MLST pattern	Allelic type							Source^a^				
			
			*aroC*	*dnaN*	*hemD*	*hisD*	*purE*	*sucA*	*thrA*	CSBS	CSAS	HS	FS	SS
Group O:4 (B) (*n* = 55)	Typhimurium	ST19 (*n* = 40)	10	7	12	9	5	9	2	6/40 (15%)	8 (20%)	5 (12.5%)	18 (45%)	3 (7.5%)
		ST34 (*n* = 14)	10	19	12	9	5	9	2	2 (14.29%)	2 (14.29%)	10 (71.43%)	0 (0%)	0 (0%)
	Derby	ST40 (*n* = 1)	19	20	3	20	5	22	22	0 (0%)	1 (100%)	0 (0%)	0 (0%)	0 (0%)
Group O:3,10 (E1) (*n* = 14)	London	ST155 (*n* = 14)	10	60	58	66	6	65	16	11 (78.57%)	1 (7.14%)	0 (0%)	2 (14.29%)	0 (0%)
Group O:7 (C1) (*n* = 7)	Rissen	ST469 (*n* = 7)	92	107	79	156	64	151	87	0 (0%	3 (42.86%)	1 (14.29%)	0 (0%)	3 (42.86%)
Group O:8 (C2-C3) (*n* = 2)	Goldcoast	ST358 (*n* = 2)	5	110	35	122	2	19	22	0 (0%)	0 (0%)	0 (0%)	2 (100%)	0 (0%)

*^*a*^CSBS, carcass swabs before splitting; CSAS, carcass swabs after splitting; HS, hepatobiliary samples; FS, fecal samples; SS, sewer samples.*

### Phenotypic Antimicrobial Resistance

**FIGURE 1:**
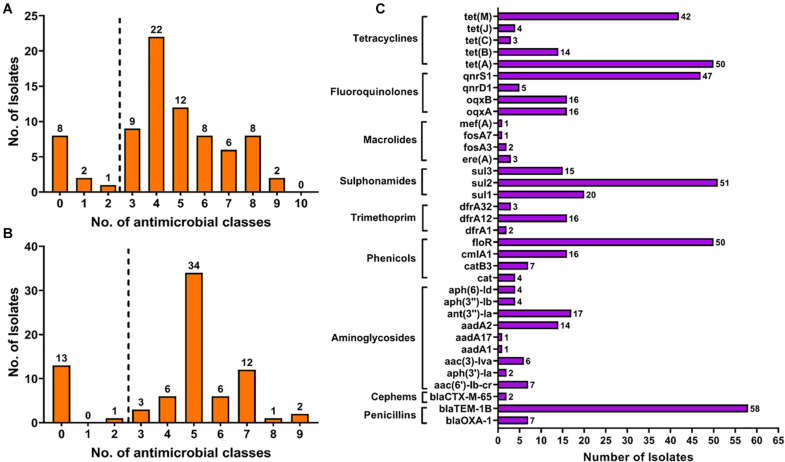
The distribution of multiple drug resistance isolates according to the results obtained by phenotypical **(A)** and genotypical **(B)** tests. The detection of antimicrobial resistance genes **(C)** showed the high prevalence of resistance gene encoding resistance to penicillins, phenicols, sulfonamides, fluoroquinolones, and tetracyclines.

The antimicrobial resistance of the isolated *Salmonella* strains was evaluated against 14 antimicrobial agents belonging to 10 classes or categories. The phenotypic antimicrobial profiles were classified as resistant, susceptible, and intermediate according to the criteria of the Clinical Laboratory Standard Institute guidelines and the results are presented in Table 4 and Supplementary Material 1. Our findings showed that tetracycline (85.90%; 67/78) and ampicillin (84.62%; 66/78) were the most resistant antimicrobial agent, followed by chloramphenicol (71.80%; 56/78) and nalidixic acid (61.54%; 48/78). Additionally, after considering the results of intermediate resistance as resistant strains, our findings showed that 89.74% (70/78) of isolates were resistant at least to one antimicrobial class, 87.18% (68/78) were resistant to at least two antimicrobial classes, and 85.90% (67/78) were resistant to at least three antimicrobial classes and were considered as MDR (Figure 1A).

**TABLE 4:** Antimicrobial susceptibility interpretation of the isolated *Salmonella* strains (*n* = 78).

Antibiotic agent	Abbreviation	Breakpoint interpretive criteria (μg/ml)^a^			Results in percentage (%)		
			
		S	I	R	S	I	R
**Penicillin:**							
Ampicillin	AMP	≤ 8	16	≥ 32	15.38% (12/78)	0% (0/78)	84.62% (66/78)
**β-Lactam combination:**							
Amoxicillin/clavulanic acid	AMC	≤ 8/4	16/8	≥ 32/16	78.21% (61/78)	21.79% (17/78)	0% (0/78)
**Cephems:**							
Cefoxitin	CX	≤ 8	16	≥ 32	98.72% (77/78)	1.28% (1/78)	0% (0/78)
Ceftiofur	CF	≤ 2	4	≥ 8	96.15% (75/78)	1.28% (1/78)	2.56% (2/78)
**Aminoglycosides:**							
Gentamicin	GEN	≤ 4	8	≥ 16	92.31% (72/78)	1.28% (1/78)	6.41% (5/78)
Kanamycin	KAN	≤ 16	32	≥ 64	89.74% (70/78)	1.28% (1/78)	8.97% (7/78)
Streptomycin^*b*^	STR	≤ 8	16	≥ 32	67.95% (53/78)	10.26% (8/78)	21.79% (17/78)
**Fluoroquinolones:**							
Ciprofloxacin	CIP	≤ 0.06	0.12–0.5	≥ 1	73.08% (57/78)	19.23% (15/78)	7.69% (6/78)
Nalidixic acid	NAL	≤ 16	–	≥ 32	38.46% (30/78)	–	61.54% (48/78)
**Tetracyclines:**							
Tetracycline	TET	≤ 4	8	≥ 16	14.10% (11/78)	0% (0/78)	85.90% (67/78)
**Phenicols:**							
Chloramphenicol	CHL	≤ 8	16	≥ 32	25.64% (20/78)	2.56% (2/78)	71.80% (56/78)
**Macrolide:**							
Azithromycin	AZI	≤ 16	–	≥ 32	93.59% (73/78)	–	6.41% (5/78)
**Polymyxins:**							
Colistin	COL	≤ 2	–	≥ 4	78.21% (61/78)	–	21.79% (17/78)
**Folate pathway inhibitors:**							
Trimethoprim/sulfamethoxazole	TST	≤ 2/38	–	≥ 4/76	55.13% (43/78)	–	44.87% (35/78)

*^*a*^S, sensitive; I, intermediate resistance; and R, resistant.*

*^*b*^For streptomycin, we used the same MIC breakpoints as for netilmicin.*

**FIGURE 2:**
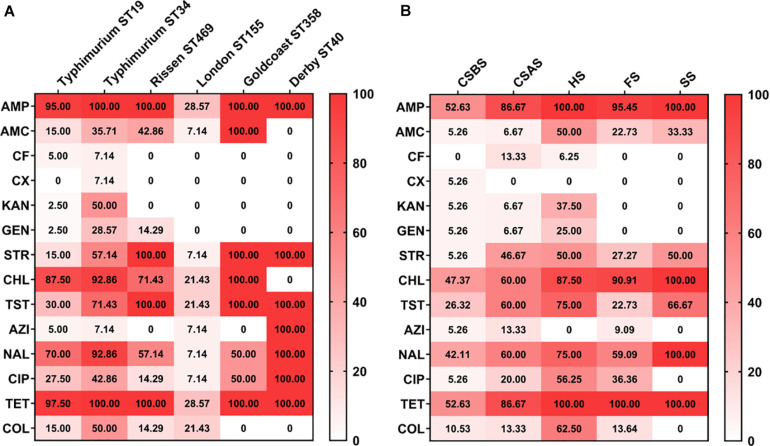
Heatmap of antimicrobial resistance of *Salmonella* isolated from pig slaughtering process according to serovars and sampling sources. The isolates of *Salmonella* Typhimurium ST34 were resistant to all the tested antimicrobial agents **(A)**, while *Salmonella* isolated from HS were the most resistant to the tested antimicrobial agents **(B)**. The numbers in cells correspond to the percentage (%) of antimicrobial resistance isolates. CSBS, carcass swabs before splitting; CSAS, carcass swabs after splitting; HS, hepatobiliary samples; FS, fecal samples; SS, sewer samples.

According to the sources, it appears that *Salmonella* isolates recovered from sewer samples (SS) and hepatobiliary samples (HS) were more resistant to the tested antimicrobial agents compared with those collected from other sources (Figure 2B). Moreover, among different serovars identified in this study, *Salmonella* serovars Derby and Goldcoast appear to be the most resistant to the tested antimicrobial agents (Figure 2A). However, it should be noted that this conclusion cannot be generalized since only one strain of *Salmonella* Derby and two strains of *Salmonella* Goldcoast have been identified in this study. Additionally, *Salmonella* Typhimurium isolates from ST34 appear to be more resistant than isolates from ST19 (Figure 2A).

### Antimicrobial Resistance Gene Prediction

**FIGURE 3:**
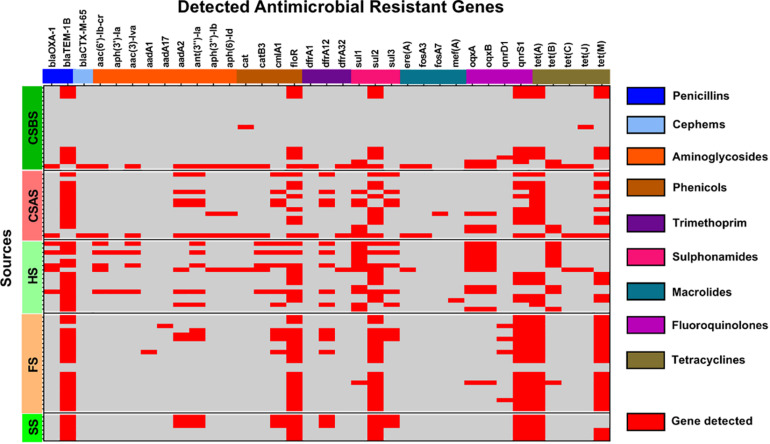
Heatmap of the detection of antimicrobial resistance genes among the studied *Salmonella* isolates (*n* = 78).

**FIGURE 4:**
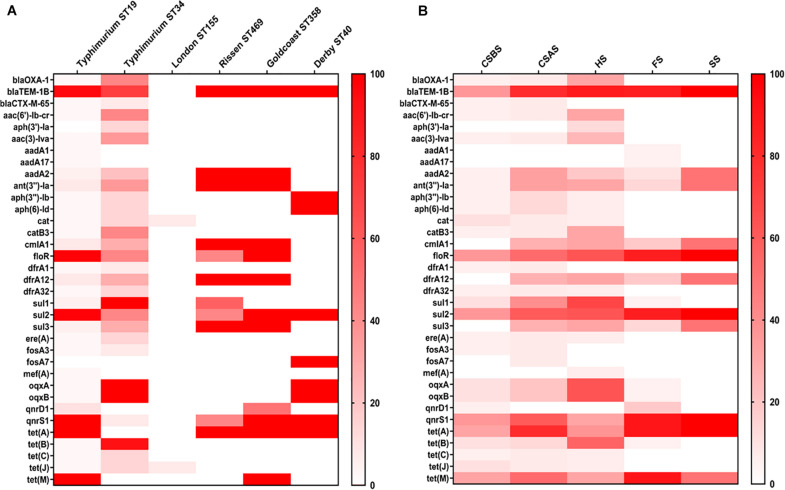
Heatmap of antimicrobial resistance genes according to serovars **(A)** and sampling sources **(B)**. The isolates of *Salmonella* Typhimurium ST34 harbor the most diversified antimicrobial resistance genes. However, *Salmonella* isolates recovered from HS and CSAS contain more resistance genes compared with those isolated from other sources. CSBS, carcass swabs before splitting; CSAS, carcass swabs after splitting; HS, hepatobiliary samples; FS, fecal samples; SS, sewer samples.

The whole genome sequences of the 78 isolated *Salmonella* strains were subjected to *in silico* detection of antimicrobial resistance genes. The results obtained showed the detection of 35 different genes encoding resistance to nine antimicrobial classes (Figure 3 and Supplementary Material 2). The most detected genes were *bla*_*TEM–1B*_ encoding resistance to penicillins (74.36%; 58/78), *sul2* encoding resistance to sulfonamides (87.93%; 51/58), *tet(A)* encoding resistance to tetracyclines (64.10%; 50/78), *floR* encoding resistance to phenicols (64.10%; 50/78), and *qnrS1* encoding resistance to fluoroquinolones (60.26%; 47/78) (Figure 1C). Moreover, 64 of 78 isolates (82.05%) harbor the resistance genes of more than two classes (Figure 1B). However, regarding the serovar distribution, it appears that *Salmonella* Typhimurium ST34 harbors more diversified antimicrobial resistance genes while *Salmonella* London ST155 appears to be poor in resistance genes (only one strain that harbors the genes *cat* and *tet(J)* encoding resistance to phenicols and tetracyclines classes, respectively) (Figure 4A). Moreover, our results showed that *Salmonella* isolates obtained from carcass swabs after splitting (CSAS) and HS harbor more resistance genes compared with those isolated from other sources (Figure 4B).

The relation between phenotypical antimicrobial resistance and the presence/absence of corresponding resistance gene obtained by *in silico* analysis was evaluated and the results are presented in Table 5. Our results showed that ciprofloxacin has the higher incoherence percentage (55.13%; 43/78) for which several isolates were genotypically positive but phenotypically negative, while cefoxitin presents the lower incoherence percentage (1.28%; 1/78).

**TABLE 5:** Phenotypical and genotypical analyses of antimicrobial resistance of *Salmonella* isolates.

Antimicrobial class	Antimicrobial agent	Coherent results		Incoherent results		Percentage of incoherence
			
		Both resistant	Both susceptible	Phenotype resistant and Genotype susceptible	Genotype resistant and Phenotype susceptible	
Penicillins	Ampicillin	60	11	6	1	8.97% (7/78)
Cephems	Ceftiofur	1	74	2	1	3.85% (3/78)
	Cefoxitin	1	76	0	1	1.28% (1/78)
Aminoglycosides	Kanamycin	7	56	1	14	19.23% (15/78)
	Gentamycin	6	57	0	15	19.23% (15/78)
	Streptomycin	20	52	5	1	7.69% (6/78)
Phenicols	Chloramphenicol	48	13	10	7	21.79% (17/78)
Macrolides	Azithromycin	3	71	2	2	5.13% (4/78)
Fluoroquinolones	Nalidixic acid	48	16	2	12	17.95% (14/78)
	Ciprofloxacin	19	16	2	41	55.13% (43/78)
Tetracyclines	Tetracycline	64	10	3	1	5.13% (4/78)
Polymyxins	Colistin	0	61	17	0	21.79% (17/78)

### Virulence Gene Prediction

In this study, the presence of 117 genes that are implicated in virulence and pathogenicity mechanisms of *Salmonella* was evaluated among the genomes of the 78 *Salmonella* isolates. The results are summarized in Supplementary Material 3. Our results showed that the number of detected genes ranged from 88 to 113 per isolate. Among the 78 isolates, three isolates (two *Salmonella* Goldcoast ST358 and one *Salmonella* Typhimurium ST19) were positive for the gene *cdtB* encoding typhoid toxin production, and these isolates were all isolated from fecal samples (FS). Additionally, only one *Salmonella* isolate (*Salmonella* London ST155) isolated from CSBS sample was positive for the genes encoding for the siderophore “yersiniabactin” (*fyuA*, *ybtA*, *ybtE*, *ybtP*, *ybtQ*, *ybtS*, *ybtT*, *ybtU*, *ybtX*, *irp1*, and *irp2*) and for the gene *senB* encoding for enterotoxin production. However, the typical virulence factors carried on *Salmonella* Pathogenicity Island 1 and 2 (SPI-1 and SPI-2) were detected in all the studied isolates.

### Plasmid Profiles

**FIGURE 5:**
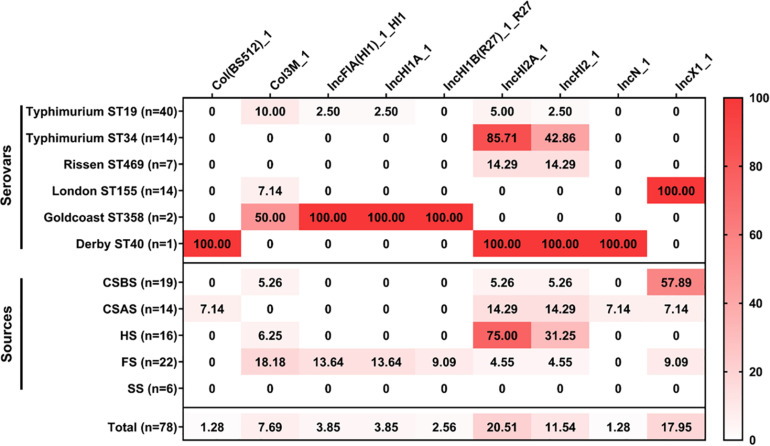
Heatmap of plasmid replicon distribution in the studied *Salmonella* isolates (*n* = 78). The numbers in cells correspond to the prevalence (%) of plasmid replicons in *Salmonella* isolates according to serovar distribution and sampling sources.

The results of the prediction of plasmid replicons in the 78 *Salmonella* isolates are presented in Figure 5 and Supplementary Material 4. Our results showed that the most abundant plasmid replicon was IncHI2A_1 (20.51%; 16/78), followed by IncX1_1 (17.95%; 14/78) and IncHI2_1 (11.54%; 9/78). The number of plasmid replicons ranged from 1 to 4 per isolate, while 42 of 78 (53.85%) *Salmonella* isolates do not harbor any plasmid. Regarding serovars, our results showed that *Salmonella* Typhimurium ST19 had a large number of different plasmids replicons (five plasmids), followed by *Salmonella* Goldcoast ST358 and *Salmonella* Derby ST40 (four plasmids). However, regarding the sampling sources, our results showed that the isolates recovered from FS harbor a large number of plasmid replicons (seven types of plasmids), followed by those recovered from CSAS (five types of plasmids), while *Salmonella* isolates recovered from SS do not harbor any plasmid.

## Discussion

Pig slaughterhouses are critical points of the meat processing chain; they are situated downstream of the pig-breeding process and upstream of pork sales. Since reception, animals undergo different complicated manipulations and are in contact with slaughterhouse facilities, workers, etc., which favors the contamination/cross-contamination of animal carcasses and, thus, the meat products (Arguello et al., 2013; Zhou et al., 2018). However, comparison between the contamination rate of *Salmonella* in pigs in the preslaughter stage and in the postslaughter stage revealed that the prevalence in the preslaughter stage often seems to be lower (Jiang et al., 2019). In this regard, Colello and his group conducted a study along the production chain of pig farms and showed that the prevalence of *Salmonella* in farms (2.6%) and slaughterhouses (2.0%) was lower than that observed in boning rooms (8.8%) and retail markets (8.0%) (Colello et al., 2018). Additionally, Jiang et al. reported that the prevalence of *Salmonella* in pigs at the farm stage was 11.77%, lower than that observed in the slaughtered pigs (45.23%) (Jiang et al., 2019), demonstrating the criticality of the slaughtering process in determining the quality and safety of derived pig food products.

In this regard, we conducted a study to evaluate the prevalence of *Salmonella* during the pig slaughtering process. Our results showed that 55 of the 226 samples (24.37%) were contaminated by *Salmonella*. These results were lower than those reported previously in pig slaughterhouses in other Chinese regions (ranged between 29.2 and 46.6%) (Bai et al., 2015; Li et al., 2016; Zhou et al., 2017) and in Spain (39.7%) (Arguello et al., 2012), while they were higher than those reported in the slaughtered pigs in Sardinia, Italy (12.9%) (Fois et al., 2017); pig carcasses and intestines from five slaughterhouses in Belgium (14.1%) (De Busser et al., 2011); a pig slaughterhouse in Yangzhou, China (17.43%) (Li et al., 2019); pork and slaughterhouse environment in Ahmedabad, Gujarat, India (13.7%) (Chaudhary et al., 2015); and pig slaughterhouses in two different regions of southwestern Spain (12.93%) (Morales-Partera et al., 2018). According to the slaughtering process, samples recovered from the dehairing area and splitting area were the most contaminated samples. In the dehairing area, the frequently used knife for carcass modification was considered as the risk factor for the observed carcass cross-contamination. However, the splitting step located at the next step after evisceration has been confirmed as the other step with a higher risk of *Salmonella* contamination (Cai et al., 2016; Zhou et al., 2017). After evisceration, the intestinal content can contaminate a part of animal carcasses; however, during splitting, the splitter could be contaminated and then cross-contaminate other carcasses, resulting in the increase of *Salmonella* prevalence in the splitting area (Cai et al., 2016; Li et al., 2016). Therefore, the implementation of good hygienic practices and management systems to control critical points during the slaughtering process is of high priority to reduce the prevalence of *Salmonella*.

Among the 55 positive samples, 78 different *Salmonella* isolates were identified in this study. These isolates belong to five different serovars and six MLST patterns, namely, with importance degree, *Salmonella* Typhimurium ST19, *Salmonella* Typhimurium ST34, *Salmonella* London ST155, *Salmonella* Rissen ST469, *Salmonella* Goldcoast ST358, and *Salmonella* Derby ST40. In China, *Salmonella* Derby was identified as the most isolated serovar from pig slaughterhouse samples (Li et al., 2016, 2019; Zhou et al., 2017, 2018; Liu et al., 2020). However, *Salmonella* Typhimurium has been reported previously as the dominant serovar in *Salmonella* isolates recovered from pig slaughterhouses in Henan Province (Bai et al., 2015). In fact, it is well known that *Salmonella* Typhimurium was classified among the major serovars causing human salmonellosis worldwide (CDC, 2018; EFSA and ECDC, 2018), especially those with multilocus sequence types ST19 and ST34, which were reported in several cases of human infections (Wong et al., 2013; Carden et al., 2015; Panzenhagen et al., 2018; Luo et al., 2020; Monte et al., 2020). Therefore, the transmission of these isolates to the final meat products along the food chain is of high risk for consumers and may cause severe cases of foodborne diseases.

The infections caused by *Salmonella* are treated with different antimicrobial drugs. However, in the last decades, development of *Salmonella* resistance to many antimicrobials has been observed worldwide, either for the isolates provided from clinical, food, and environmental samples. In this study, the phenotypical and genotypical antimicrobial resistance profiles of the 78 isolated *Salmonella* strains were evaluated. Phenotypical results classified tetracycline and ampicillin as the less effective antimicrobial agents. In fact, the high resistance of *Salmonella* isolates to tetracycline and ampicillin has been reported over the world in samples collected along the animal food chain (Ed-Dra et al., 2017; Jiang et al., 2019, 2021; Wang et al., 2019; Liu et al., 2020, 2021), since they were frequently used in animal farms (Lekagul et al., 2019). In fact, the abuse and the misuse of antimicrobial drugs in animal livestock for therapeutic, prophylaxis, and growth promotions have led to the development of antimicrobial resistance. Moreover, our results showed that 85.90% of isolates/strains were resistant to more than two antimicrobial classes (MDR), which is considered a serious threat to public health that leads to therapeutic failure after a simple infection by MDR isolates.

Genotypical antimicrobial resistance prediction showed the detection of 35 resistance genes encoding resistance to nine antimicrobial classes, with a high prevalence of *bla*_TEM–1B_ gene encoding resistance to penicillins, *sul2* gene encoding resistance to sulfonamides, *tet(A)* gene encoding resistance to tetracyclines, *floR* gene encoding resistance to phenicols, and *qnrS1* gene encoding resistance to fluoroquinolone. The presence of these genes in bacterial genomes could be responsible for the acquisition of resistance to the corresponding antimicrobial classes. However, the analysis of coherence between genotypic and phenotypic antimicrobial resistance showed that phenotypic resistance cannot always be linked to the presence of resistance genes. Our results are in agreement with those reported previously in *Salmonella* isolates, showing a difference between phenotypic and genotypic resistance profiles (Liu et al., 2020, 2021). Hence, the phenotypic test remains the gold method for the assessment of bacterial behavior toward antimicrobial agents.

The prediction of virulence genes implicated in virulence and pathogenicity mechanisms reveals the detection of 117 different genes, particularly the detection of *cdtB* gene encoding typhoid toxins in two isolates of *Salmonella* Goldcoast ST358 and one isolate of *Salmonella* Typhimurium ST19 and the detection of genes encoding for the siderophore “yersiniabactin” in one isolate of *Salmonella* London ST155, and this isolate also harbors the gene encoding for the enterotoxin TieB (*senB*). In fact, it has been reported that the presence of *cdtB* in the *Salmonella* genome was linked to isolates implicated in human bloodstream and invasive infections (Miller et al., 2018; Xu X. et al., 2020). Additionally, yersiniabactin siderophore that was initially described in *Yersinia* spp. is required for iron uptake and growth of the bacteria in an iron-restricted environment (Perry and Fetherston, 2011; Khan et al., 2018). However, the enterotoxin TieB was initially described in enteroinvasive *E. coli* (EIEC) (Nataro et al., 1995) and has been suggested to play a key role in bacteria virulence in humans (Meza-Segura et al., 2020). Indeed, the presence of these virulence genes in the genome of *Salmonella* isolated from the pig slaughtering process may lead to severe disease outcomes in humans.

In this study, nine different plasmid replicons were detected among the 78 *Salmonella* isolates. The most abundant plasmids were IncHI2A_1, IncX1_1, and IncHI2_1. IncHI2A_1 and IncHI2_1 were predominant in *Salmonella* Typhimurium ST34, while IncX1_1 was detected only in *Salmonella* London ST155. These plasmids were identified previously in *Salmonella* isolates recovered from the animal food chain, especially pork production chains (Liu et al., 2020, 2021; Viana et al., 2020). Interestingly, it has been demonstrated that these plasmids were associated with resistance to different antimicrobial classes, including β-lactams, aminoglycosides, sulfonamides, tetracyclines, and polymyxins (Elbediwi et al., 2020b,a; Gu et al., 2020; McMillan et al., 2020). Consequently, these plasmids may mediate the horizontal transmission of antimicrobial resistance genes during this slaughtering process.

## Conclusion

In this study, we provided the dynamic prevalence of *Salmonella* during the pig slaughtering process. Additionally, we demonstrated the use of whole genome sequencing as a cost-effective approach for routine surveillance of foodborne pathogens, especially *Salmonella*. The prediction of serovar distribution, MLST patterns, antimicrobial resistance genes, plasmid replicons, and virulence factors in *Salmonella* isolates recovered from the pig slaughtering process showed the isolation of MDR isolates harboring different antimicrobial resistant determinants and virulence factors like *cdtB* gene encoding typhoid toxins, *senB* gene encoding for the enterotoxin production, and several genes encoding for the siderophore “yersiniabactin.” Therefore, it is time to prevent the use of antimicrobials in animal livestock in order to avoid the dissemination of antimicrobial resistance determinants along the food chain and to implement management systems to control critical points in order to avoid the transmission of foodborne pathogens to humans.

## Data Availability Statement

The datasets generated for this study can be found in the NCBI Bioproject with the accession number no. PRJNA686895.

## Author Contributions

BW, AE-D, and HP contributed equally to this work. AE-D and HP analyzed the data and finalized the figures. AE-D and MY were wrote the manuscript. BW, HP, CD, and CJ did the experiment and data collection. MY conceived the idea and assisted with data analysis and writing. All authors read, revised, and approved the final manuscript.

## Conflict of Interest

The authors declare that the research was conducted in the absence of any commercial or financial relationships that could be construed as a potential conflict of interest.

## Footnotes

1 https://githubcom/tseemann/mlst2016

## References

[B1] Afgan E. Baker D. van den Beek M. Blankenberg D. Bouvier D. Čech M. (2016). The Galaxy platform for accessible, reproducible and collaborative biomedical analyses: 2016 update. *Nucleic Acids Res.* 44 W3–W10. 10.1093/nar/gkw343 27137889 PMC4987906

[B2] Arguello H. Álvarez-Ordoñez A. Carvajal A. Rubio P. Prieto M. (2013). Role of slaughtering in *Salmonella* spreading and control in pork production. *J. Food Prot.* 76 899–911. 10.4315/0362-028X.JFP-12-404 23643137

[B3] Arguello H. Carvajal A. Collazos J. A. García-Feliz C. Rubio P. (2012). Prevalence and serovars of *Salmonella enterica* on pig carcasses, slaughtered pigs and the environment of four Spanish slaughterhouses. *Food Res. Int.* 45 905–912. 10.1016/j.foodres.2011.04.017

[B4] Asai T. Namimatsu T. Osumi T. Kojima A. Harada K. Aoki H. (2010). Molecular typing and antimicrobial resistance of *Salmonella enterica* subspecies enterica serovar Choleraesuis isolates from diseased pigs in Japan. *Comp. Immunol. Microbiol. Infect. Dis.* 33 109–119. 10.1016/j.cimid.2008.08.004 18809209

[B5] Bai L. Lan R. Zhang X. Cui S. Xu J. Guo Y. (2015). Prevalence of *Salmonella* isolates from chicken and pig slaughterhouses and emergence of ciprofloxacin and cefotaxime co-resistant *S. enterica* serovar Indiana in Henan, China. *PLoS One* 10:144532. 10.1371/journal.pone.0144532 26650239 PMC4674084

[B6] Bankevich A. Nurk S. Antipov D. Gurevich A. A. Dvorkin M. Kulikov A. S. (2012). SPAdes: a new genome assembly algorithm and its applications to single-cell sequencing. *J. Comput. Biol.* 19 455–477. 10.1089/cmb.2012.0021 22506599 PMC3342519

[B7] Besser J. M. (2018). *Salmonella* epidemiology: a whirlwind of change. *Food Microbiol.* 71 55–59. 10.1016/j.fm.2017.08.018 29366469

[B8] Biasino W. De Zutter L. Mattheus W. Bertrand S. Uyttendaele M. Van Damme I. (2018). Correlation between slaughter practices and the distribution of *Salmonella* and hygiene indicator bacteria on pig carcasses during slaughter. *Food Microbiol.* 70 192–199. 10.1016/j.fm.2017.10.003 29173627

[B9] Biswas S. Elbediwi M. Gu G. Yue M. (2020). Genomic characterization of new variant of hydrogen sulfide (H2S)-producing *escherichia coli* with multidrug resistance properties carrying the mcr-1 gene in China. *Antibiotics* 9:80. 10.3390/antibiotics9020080 32069849 PMC7167817

[B10] Bonardi S. (2017). *Salmonella* in the pork production chain and its impact on human health in the European Union. *Epidemiol. Infect.* 145 1513–1526. 10.1017/S095026881700036X 28241896 PMC9203350

[B11] Boyen F. Haesebrouck F. Maes D. Van Immerseel F. Ducatelle R. Pasmans F. (2008). Non-typhoidal *Salmonella* infections in pigs: a closer look at epidemiology, pathogenesis and control. *Vet. Microbiol.* 130 1–19. 10.1016/j.vetmic.2007.12.017 18243591

[B12] Cai Y. Tao J. Jiao Y. Fei X. Zhou L. Wang Y. (2016). Phenotypic characteristics and genotypic correlation between *Salmonella* isolates from a slaughterhouse and retail markets in Yangzhou, China. *Int. J. Food Microbiol.* 222 56–64. 10.1016/j.ijfoodmicro.2016.01.020 26851738

[B13] Carattoli A. Zankari E. Garciá-Fernández A. Larsen M. V. Lund O. Villa L. (2014). In Silico detection and typing of plasmids using plasmidfinder and plasmid multilocus sequence typing. *Antimicrob. Agents Chemother.* 58 3895–3903. 10.1128/AAC.02412-14 24777092 PMC4068535

[B14] Carden S. Okoro C. Dougan G. Monack D. (2015). Non-typhoidal *Salmonella* Typhimurium ST313 isolates that cause bacteremia in humans stimulate less inflammasome activation than ST19 isolates associated with gastroenteritis. *Pathog. Dis.* 73:ftu023. 10.1093/femspd/ftu023 25808600 PMC4399442

[B15] CDC (2018). *National Enteric Disease Surveillance: Salmonella* Annual Report, 2016. Available online at: https://www.census.gov/geo/pdfs/maps-data/maps/ (Accessed April 24, 2021)

[B16] Chaudhary J. H. Nayak J. B. Brahmbhatt M. N. Makwana P. P. (2015). Virulence genes detection of *Salmonella* serovars isolated from pork and slaughterhouse environment in Ahmedabad, Gujarat. *Vet. World* 8 121–124. 10.14202/vetworld.2015.121-124 27047008 PMC4777800

[B17] Chen L. Yang J. Yu J. Yao Z. Sun L. Shen Y. (2005). VFDB: a reference database for bacterial virulence factors. *Nucleic Acids Res.* 33 D325–D328. 10.1093/nar/gki008 15608208 PMC539962

[B18] CLSI (2017). *Performance Standards for Antimicrobial Susceptibility Testing*, 27th Edn. Wayne, PA: Clinical and Laboratory Standards Institute.

[B19] Colello R. Ruiz M. J. Padín V. M. Rogé A. D. Leotta G. Padola N. L. (2018). Detection and characterization of *Salmonella* Serotypes in the production chain of two pig farms in Buenos Aires Province, Argentina. *Front. Microbiol.* 9:1370. 10.3389/fmicb.2018.01370 30002649 PMC6031755

[B20] De Busser E. V. Maes D. Houf K. Dewulf J. Imberechts H. Bertrand S. (2011). Detection and characterization of *Salmonella* in lairage, on pig carcasses and intestines in five slaughterhouses. *Int. J. Food Microbiol.* 145 279–286. 10.1016/j.ijfoodmicro.2011.01.009 21276632

[B21] Deen J. von Seidlein L. Andersen F. Elle N. White N. J. Lubell Y. (2012). Community-acquired bacterial bloodstream infections in developing countries in south and southeast Asia: a systematic review. *Lancet Infect. Dis.* 12 480–487. 10.1016/S1473-3099(12)70028-2 22632186

[B22] Ed-Dra A. Filali F. R. Karraouan B. El Allaoui A. Aboulkacem A. Bouchrif B. (2017). Prevalence, molecular and antimicrobial resistance of *Salmonella* isolated from sausages in Meknes, Morocco. *Microb. Pathog.* 105 340–345. 10.1016/j.micpath.2017.02.042 28258000

[B23] Ed-Dra A. Karraouan B. El Allaoui A. Khayatti M. El Ossmani H. Rhazi Filali F. (2018). Antimicrobial resistance and genetic diversity of *Salmonella* Infantis isolated from foods and human samples in Morocco. *J. Glob. Antimicrob. Resist.* 14 297–301. 10.1016/j.jgar.2018.05.019 29842977

[B24] EFSA, and ECDC (2018). The European Union summary report on trends and sources of zoonoses, zoonotic agents and food-borne outbreaks in 2017. *EFSA J.* 16:e05500. 10.2903/j.efsa.2018.5500 32625785 PMC7009540

[B25] Elbediwi M. Pan H. Biswas S. Li Y. Yue M. (2020a). Emerging colistin resistance in *Salmonella enterica* serovar Newport isolates from human infections. *Emerg. Microbes Infect.* 9 535–538. 10.1080/22221751.2020.1733439 32122270 PMC7067173

[B26] Elbediwi M. Pan H. Jiang Z. Biswas S. Li Y. Yue M. (2020b). Genomic characterization of mcr-1-carrying *Salmonella enterica* Serovar 4,[5],12:i:- ST 34 clone isolated from pigs in China. *Front. Bioeng. Biotechnol.* 8:663. 10.3389/fbioe.2020.00663 32714906 PMC7344297

[B27] Fois F. Piras F. Torpdahl M. Mazza R. Consolati S. G. Spanu C. (2017). Occurrence, characterization, and antimicrobial susceptibility of *Salmonella enterica* in slaughtered pigs in Sardinia. *J. Food Sci.* 82 969–976. 10.1111/1750-3841.13657 28226178

[B28] Gu D. Wang Z. Tian Y. Kang X. Meng C. Chen X. (2020). Prevalence of *Salmonella* isolates and their distribution based on whole-genome sequence in a chicken slaughterhouse in Jiangsu, China. *Front. Vet. Sci.* 7:29. 10.3389/fvets.2020.00029 32154275 PMC7046563

[B29] Jiang Z. Anwar T. M. Peng X. Biswas S. Elbediwi M. Li Y. (2021). Prevalence and antimicrobial resistance of *Salmonella* recovered from pig-borne food products in Henan, China. *Food Control* 121:107535. 10.1016/j.foodcont.2020.107535

[B30] Jiang Z. Paudyal N. Xu Y. Deng T. Li F. Pan H. (2019). Antibiotic resistance profiles of *Salmonella* recovered from finishing pigs and slaughter facilities in Henan, China. *Front. Microbiol.* 10:1513. 10.3389/fmicb.2019.01513 31333618 PMC6622357

[B31] Jun W. ZengRen Z. JingYu W. (2007). Risk assessment of *Salmonella* in animal derived food. *Chin. J. Anim. Quar.* 24 23–25.

[B32] Kagambèga A. Lienemann T. Aulu L. Traoré A. S. Barro N. Siitonen A. (2013). Prevalence and characterization of *Salmonella enterica* from the feces of cattle, poultry, swine and hedgehogs in Burkina Faso and their comparison to human *Salmonella* isolates. *BMC Microbiol.* 13:253. 10.1186/1471-2180-13-253 24215206 PMC3828578

[B33] Khan A. Singh P. Srivastava A. (2018). Synthesis, nature and utility of universal iron chelator–Siderophore: a review. *Microbiol. Res.* 212–213 103–111. 10.1016/j.micres.2017.10.012 29103733

[B34] Lekagul A. Tangcharoensathien V. Yeung S. (2019). Patterns of antibiotic use in global pig production: a systematic review. *Vet. Anim. Sci.* 7:100058. 10.1016/j.vas.2019.100058 32734079 PMC7386699

[B35] Lettini A. A. Vo Than T. Marafin E. Longo A. Antonello K. Zavagnin P. (2016). Distribution of *Salmonella* serovars and antimicrobial susceptibility from poultry and swine farms in central Vietnam. *Zoonoses Public Health* 63 569–576. 10.1111/zph.12265 26952244

[B36] Li Q. Yin J. Li Z. Li Z. Du Y. Guo W. (2019). Serotype distribution, antimicrobial susceptibility, antimicrobial resistance genes and virulence genes of *Salmonella* isolated from a pig slaughterhouse in Yangzhou, China. *AMB Express* 9:210. 10.1186/s13568-019-0936-9 31884559 PMC6935380

[B37] Li R. Lai J. Wang Y. Liu S. Li Y. Liu K. (2013). Prevalence and characterization of *Salmonella* species isolated from pigs, ducks and chickens in Sichuan Province, China. *Int. J. Food Microbiol.* 163 14–18. 10.1016/j.ijfoodmicro.2013.01.020 23474653

[B38] Li Y. Cai Y. Tao J. Kang X. Jiao Y. Guo R. (2016). *Salmonella* isolated from the slaughterhouses and correlation with pork contamination in free market. *Food Control* 59 591–600. 10.1016/j.foodcont.2015.06.040

[B39] Liu Q. Chen W. Elbediwi M. Pan H. Wang L. Zhou C. (2020). Characterization of *Salmonella* resistome and plasmidome in pork production system in Jiangsu, China. *Front. Vet. Sci.* 7:617. 10.3389/FVETS.2020.00617 33062654 PMC7517575

[B40] Liu Y. Jiang J. Ed-Dra A. Li X. Peng X. Xia L. (eds). (2021). Prevalence and genomic investigation of *Salmonella* isolates recovered from animal food-chain in Xinjiang, China. *Food Res. Int.* 142:110198. 10.1016/j.foodres.2021.110198 33773671

[B41] Luo Q. Wan F. Yu X. Zheng B. Chen Y. Gong C. (2020). MDR *Salmonella enterica* serovar Typhimurium ST34 carrying mcr-1 isolated from cases of bloodstream and intestinal infection in children in China. *J. Antimicrob. Chemother.* 75 92–95. 10.1093/jac/dkz415 31580437

[B42] Mao X. Hu J. Liu X. (2011). Estimation on disease burden of foodborne non-typhoid salmonellosis in China using literature review method. *Chin. J. Dis. Control Prev.* 15 622–625.

[B43] McMillan E. A. Jackson C. R. Frye J. G. (2020). Transferable plasmids of *Salmonella enterica* associated with antibiotic resistance genes. *Front. Microbiol.* 11:562181. 10.3389/fmicb.2020.562181 33133037 PMC7578388

[B44] Meza-Segura M. Zaidi M. B. Vera-Ponce de León A. Moran-Garcia N. Martinez-Romero E. Nataro J. P. (2020). New insights into DAEC and EAEC pathogenesis and phylogeny. *Front. Cell. Infect. Microbiol.* 10:572951. 10.3389/fcimb.2020.572951 33178627 PMC7593697

[B45] Miller R. A. Betteken M. I. Guo X. Altier C. Duhamel G. E. Wiedmann M. (2018). The typhoid toxin produced by the Nontyphoidal *Salmonella enterica* serotype javiana is required for induction of a DNA damage response *in Vitro* and systemic spread *in Vivo*. *MBio* 9:e00467-18. 10.1128/mBio.00467-18 29588404 PMC5874915

[B46] Monte D. F. M. Sellera F. P. Lopes R. Keelara S. Landgraf M. Greene S. (2020). Class 1 integron-borne cassettes harboring *bla*_CARB–2_ gene in multidrug-resistant and virulent *Salmonella* Typhimurium ST19 strains recovered from clinical human stool samples, United States. *PLoS One* 15:e0240978. 10.1371/journal.pone.0240978 33125394 PMC7598458

[B47] Morales-Partera A. M. Cardoso-Toset F. Luque I. Astorga R. J. Maldonado A. Herrera-León S. (2018). Prevalence and diversity of *Salmonella* spp., *Campylobacter* spp., and *Listeria monocytogenes* in two free-range pig slaughterhouses. *Food Control* 92 208–215. 10.1016/j.foodcont.2018.04.053

[B48] Nataro J. P. Seriwatana J. Fasano A. Maneval D. R. Guers L. D. Noriega F. (1995). Identification and cloning of a novel plasmid-encoded enterotoxin of enteroinvasive *Escherichia coli* and *Shigella* strains. *Infect. Immun.* 63 4721–4728. 10.1128/iai.63.12.4721-4728.1995 7591128 PMC173677

[B49] Panzenhagen P. H. N. Paul N. C. Conte C. A. Costa R. G. Rodrigues D. P. Shah D. H. (2018). Genetically distinct lineages of *Salmonella* Typhimurium ST313 and ST19 are present in Brazil. *Int. J. Med. Microbiol.* 308 306–316. 10.1016/j.ijmm.2018.01.005 29396155

[B50] Paudyal N. Pan H. Elbediwi M. Zhou X. Peng X. Li X. (2019). Characterization of *Salmonella* Dublin isolated from bovine and human hosts. *BMC Microbiol.* 19:226. 10.1186/s12866-019-1598-0 31619165 PMC6796477

[B51] Perry R. D. Fetherston J. D. (2011). Yersiniabactin iron uptake: mechanisms and role in *Yersinia pestis* pathogenesis. *Microbes Infect.* 13 808–817. 10.1016/j.micinf.2011.04.008 21609780 PMC3148425

[B52] Rahkio M. Korkeala H. (1996). Microbiological contamination of carcasses related to hygiene practice and facilities on slaughtering lines. *Acta Vet. Scand.* 37 219–228. 10.1186/BF03548089 8996868 PMC8063981

[B53] Viana C. Grossi J. L. Sereno M. J. Yamatogi R. S. dos Santos Bersot L. Call D. R. (2020). Phenotypic and genotypic characterization of non-typhoidal *Salmonella* isolated from a Brazilian pork production chain. *Food Res. Int.* 137:109406. 10.1016/j.foodres.2020.109406 33233093

[B54] Vico J. P. Lorenzutti A. M. Zogbi A. P. Aleu G. Sánchez I. C. Caffer M. I. (2020). Prevalence, associated risk factors, and antimicrobial resistance profiles of non-typhoidal *Salmonella* in large scale swine production in Córdoba, Argentina. *Res. Vet. Sci.* 130 161–169. 10.1016/j.rvsc.2020.03.003 32193003

[B55] Wang X. Biswas S. Paudyal N. Pan H. Li X. Fang W. (2019). Antibiotic resistance in *Salmonella* Typhimurium isolates recovered from the food chain through national antimicrobial resistance monitoring system between 1996 and 2016. *Front. Microbiol.* 10:985. 10.3389/fmicb.2019.00985 31134024 PMC6514237

[B56] World Health Organisation (WHO) (2018). *Salmonella* (Non-Typhoidal). World Health Organisation. Available online at: https://www.who.int/en/news-room/fact-sheets/detail/salmonella-(non-typhoidal) (Accessed March 30, 2021)

[B57] Wilson C. N. Pulford C. V. Akoko J. Sepulveda B. P. Predeus A. V. Bevington J. (2020). *Salmonella* identified in pigs in Kenya and Malawi reveals the potential for zoonotic transmission in emerging pork markets. *PLoS Negl. Trop. Dis.* 14:e0008796. 10.1371/journal.pntd.0008796 33232324 PMC7748489

[B58] Wong M. H. Y. Yan M. Chan E. W. C. Liu L. Z. Kan B. Chen S. (2013). Expansion of *Salmonella enterica* serovar Typhimurium ST34 clone carrying multiple resistance determinants in China. *Antimicrob. Agents Chemother.* 57 4599–4601. 10.1128/AAC.01174-13 23796940 PMC3754352

[B59] Xu X. Chen Y. Pan H. Pang Z. Li F. Peng X. (2020). Genomic characterization of *Salmonella* Uzaramo for human invasive infection. *Microb. genom.* 6:mgen000401. 10.1099/mgen.0.000401 32589568 PMC7478631

[B60] Xu Y. Zhou X. Jiang Z. Qi Y. Ed-Dra A. Yue M. (eds) (2020). Epidemiological investigation and antimicrobial resistance profiles of *Salmonella* isolated from breeder chicken hatcheries in Henan, China. *Front. Cell. Infect. Microbiol.* 10:497. 10.3389/fcimb.2020.00497 33042870 PMC7522330

[B61] Yu H. Elbediwi M. Zhou X. Shuai H. Lou X. Wang H. (2020). Epidemiological and genomic characterization of *Campylobacter jejuni* isolates from a foodborne outbreak at Hangzhou, China. *Int. J. Mol. Sci.* 21:3001. 10.3390/ijms21083001 32344510 PMC7215453

[B62] Zankari E. Hasman H. Cosentino S. Vestergaard M. Rasmussen S. Lund O. (2012). Identification of acquired antimicrobial resistance genes. *J. Antimicrob. Chemother.* 67 2640–2644. 10.1093/jac/dks261 22782487 PMC3468078

[B63] Zhou Z. Jin X. Zheng H. Li J. Meng C. Yin K. (2018). The prevalence and load of *Salmonella*, and key risk points of *Salmonella* contamination in a swine slaughterhouse in Jiangsu province, China. *Food Control* 87 153–160. 10.1016/j.foodcont.2017.12.026

[B64] Zhou Z. Li J. Zheng H. Jin X. Shen Y. Lei T. (2017). Diversity of *Salmonella* isolates and their distribution in a pig slaughterhouse in Huai’an, China. *Food Control* 78 238–246. 10.1016/j.foodcont.2017.02.064

[B65] Zhu C. Yue M. Rankin S. Weill F. X. Frey J. Schifferli D. M. (2015). One-step identification of five prominent chicken *Salmonella* serovars and biotypes. *J. Clin. Microbiol.* 53 3881–3883. 10.1128/JCM.01976-15 26378281 PMC4652083

